# Understanding action on the social determinants of health: a critical realist analysis of in-depth interviews with staff of nine Ontario public health units

**DOI:** 10.1186/s13104-015-1064-5

**Published:** 2015-03-28

**Authors:** Dennis Raphael, Julia Brassolotto

**Affiliations:** School of Health Policy and Management, York University, 4700 Keele Street HNES Building, Toronto, ON M3J 1P3 Canada

**Keywords:** Social determinants of health, Public health practice, Critical realist analysis, Canada

## Abstract

**Background:**

Addressing the social determinants of health (SDH) is identified as a role for local public health units (PHUs) in the province of Ontario. Despite this authorization to do so there is wide variation in PHU practice. In this article we consider the factors that shape local PHU action on the SDH through a critical realist analysis.

**Methods:**

Interviews with Medical Officers of Health (MOHs) and lead staff from nine PHUs in Ontario identify the structures and powers that allow PHUs to address the SDH as well as the many factors that either activate or inhibit these structures and powers.

**Results:**

We found that personal backgrounds and attitudes of MOHs and leading staff people as well as local jurisdictional characteristics shape whether and how PHUs carry out SDH-related activities.

**Conclusions:**

Action on the SDH is a result of a complex interplay of micro-, meso- and macro-level factors that requires recognition of the contested nature of public health, presence of Ministry of Health mandates, local jurisdictional characteristics, and politics. The most effective way to assure PHU action on the SDH is for the Ministry of Health and Long-Term Care to mandate such activities and develop accountability mechanisms that assure implementation.

## Background

Acting on the social determinants of health (SDH) has become a critical concern of the global public health community [1]. This concern is evident in numerous statements and documents provided by international, national and local public health authorities. Despite this apparent consensus, there is wide variation among national [2] and local jurisdictions in the implementation of the SDH concept [3,4]. This variation is not due to a lack of knowledge of the importance of the SDH or the means of influencing these SDH in the service of health. Instead, we argue that this variation has much to do with differing interpretations of the public health role in addressing the SDH [5]. At the national level, these interpretations are shaped by the form of the welfare state, the politics and ideology of governing authorities, and the extent to which addressing the SDH is consistent with prevailing societal values [2]. Even when addressing the SDH is consistent with these societal values, actual implementation of the SDH concept can vary at the local level.

In jurisdictions where the SDH are on the national public health agenda, key factors that lead to successful implementation by local authorities include governing through: 1) collaboration; 2) citizen engagement; 3) a mix of regulation and persuasion; 4) independent agencies and expert bodies and 5) adaptive policies, resilient structures and foresight [6]. In jurisdictions where there is no national directive to address the SDH, implementation of the SDH concept may depend more on the initiative of local public health officials [5]. Only after a willingness to address these issues is present, can these governance issues be considered.

Canada is one of the nations where no national directive exists to address the SDH [7]. In addition, public health is a provincial responsibility and no province has explicitly placed the SDH on its broad health policy agenda. As a result, local public health unit (PHU) action is very much dependent on public health authorities’ willingness to address the SDH. In Ontario – Canada’s most populous province -- the Public Health Standards instruct local PHUs to address the SDH, but there are no concrete instructions for how to do so, nor are there accountability mechanisms to assure it happens [5,8].

**Figure 1:**
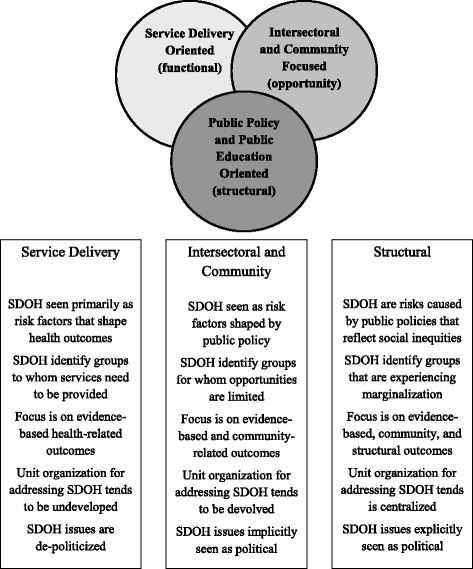
**Public health units’ differing approaches to addressing the social determinants of health.**

Our analysis of nine local PHUs in Ontario revealed three general approaches to addressing the SDH in public health practice [9] (Figure 1). *Service delivery-oriented* PHUs limited themselves to service-related activities that responded to SDH-related needs of clients while PHUs identified as *Intersectoral and Community-Focused* carried out both service-delivery and community-based intersectoral activities designed to improve services and stimulate health-promoting public policy. *Public Policy/Public Education* PHUs also carried out service delivery and engaged in policy-related community-based activities, but additionally assumed a leadership role in carrying out public policy advocacy and public education about the SDH.

We found that these differences in practice are associated with the ideological views towards addressing health inequalities held by Medical Officers of Health (MOH) and lead staff [5]. For us, an ideology constitutes a system of ideas that leads to distinct ways of viewing and responding to a problem, in this case addressing the SDH through public health practice [5].

MOHs and lead staff of service-oriented units have a functional and practical view of how SDH inform PHU practice while those in intersectoral and community-focused units have an opportunity-based analysis of the role PHUs can play in addressing the SDH. Finally, those in units carrying out public policy advocacy and public education have a structural view of society and saw the PHU role as helping to change societal structures that marginalize and exclude people. Organizational structures of PHUs parallel these ideological differences with greater centralization of SDH activities associated with more advanced activity [9]. However, our previous work did not identify how individuals’ personal backgrounds and attitudes came to shape these ideological views nor did it consider how local jurisdictional arrangements and characteristics interacted with these ideological views to shape PHUs’ SDH-related activities.

In this paper, we extend this work by considering these issues within a critical realist framework. We do so with the purpose of elucidating the existing societal structures and powers that enable SDH-related activities and identifying factors that either facilitate or prevent the activation of these structures and powers. We also consider how institutional factors interact with personal characteristics of MOHs and features of local jurisdictions to shape the form of SDH activities. This analysis provides a framework for understanding the differences in SDH activity among PHUs and contextualizing these practices within micro-, meso-, and macro-level systems of influence. It also identifies future research questions and means by which public health activity addressing the SDH can be developed and implemented.

### Structures and powers with the potential to support actions on the SDH

A critical realist perspective identifies the *real, actual*, and *empirical* levels of a phenomenon [10]. The *real* is the explication of the societal structures and powers that have the capacity to allow a phenomenon to occur. In this case, provincial guidelines, MOH responsibilities, public health association statements and collaborative working groups are the structures and powers that appear to allow PHUs to address the SDH. The *actual* refers to whether these structures and powers are activated such that a PHU carries out local SDH actions. The *actual* therefore considers how the jurisdiction’s organizational environments and characteristics, the PHU’s working environment, and the training and priorities of lead staff members either activate or inhibit these powers. The *empirical* is what the PHU actually does to address the SDH; that is, its specific programs, activities, and initiatives.

**Figure 2:**
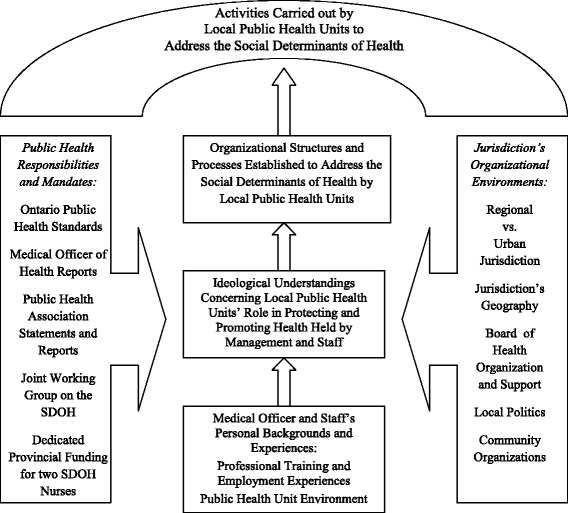
**Potential structures and processes (** ***the real*** **) as well as influences (** ***the actual*** **) upon local public health units approaches (** ***the empirical*** **) to addressing the social determinants of health.**

The value of the critical realist approach is that it reveals that powers may exist unexercised, such that what *is* happening does not preclude what *can* happen. This is especially important in the Canadian case as existing structures and powers may allow a range of SDH activities, yet translation into action is varied. This analysis can identify the barriers to be surmounted in order to enable PHU action on the SDH. Figure 2 outlines our analysis of the structures and powers (the *real*) as well as the influences that may enable these structures and processes to become enabled (the *actual*) into action (the *empirical*). As such it identifies the issues that that shape how a PHU comes to be placed in the Figure 1 typology.

The Ontario Public Health Standards mandate addressing the SDH and Ontario’s Medical Officer of Health has reported on the need to address the SDH in two Annual Reports [8,11,12]. In addition, the Ministry of Health and Long-Term Care provides dedicated funding for two public health nurses for each of the 36 PHUs to address the SDH. However, no guidance is provided on how to do this and no accountability mechanisms exist to assure implementation of SDH activities. Addressing the apparent PHU need for direction, the Ontario Public Health Association participates with the Association of Local Public Health Associations in the Joint Working Group (JWG) on the SDH [3]. Its purpose is to reduce social and health inequities by promoting local PHU activities to address the SDH. Despite JWG efforts, PHUs continue to struggle in this task [3,4].

It appears at the level of the *real,* PHUs in Ontario -- and their MOHs -- have the authority to address the SDH, but policies and practices for doing so are not mandated [8]. More advanced action on the SDH that moves beyond traditional service delivery is, however, sporadic and related to MOHs’ commitments to doing so. The following sections examine how MOHs and their lead staff come to develop these commitments and how these commitments interact with a jurisdiction’s social, political, and organizational environments to shape SDH-related activities. It is these interactions that activate or do not activate PHU powers into action.

## Methods

Details of the study’s methods are provided in greater depth elsewhere [5]. In brief, we selected a purposive maximum variation sample of nine PHUs that differed in the quantity and quality of their SDH-related activity. Three sets of information were collected and analyzed from this mix of nine regional and urban PHUs. The first set of information consisted of SDH-related printed materials and documents provided by each PHU: mission statements and reports, organizational charts and strategic plans, training manuals, and examples of public education and public advocacy. The second set of information consisted of qualitative information collected through nine structured open-ended interviews -- one for each PHU -- with seven MOHs and in two cases, the Associate MOH. The third set of information came from nine interviews with the lead staff person(s) responsible for directing and managing SDH issues for the PHU. The lead staff persons came from varied educational and professional backgrounds. Written informed consent for participation in the study was obtained from participants. There were no incentives offered for participation. Ethics approval was obtained from York University’s Ethics Board, Certificate #2011 - 086.

We conducted the interviews -- the focus of this article -- by telephone. Each interview lasted 60–90 minutes. The participants were provided with the questions in advance. Interviews were digitally recorded and transcribed. We then analyzed the data in order to understand how these differences in PHU SDH-related activity came about. We applied the constant comparison method whereby our reading of the transcripts identified repeating ideas indicative of participants’ understandings and experiences with the SDH [13,14]. These ideas were then compared and synthesized to identify key themes. Of particular interest is the relationship of findings to the typology of PHUs – service delivery oriented, intersectoral and community-focused and public advocacy/public education – identified in our earlier work.

## Results

### MOH and lead staff personal backgrounds and experiences

We inquired into the specific influences and experiences that had come to shape ways of thinking about the SDH and the public health role in addressing them. We identified three specific clusters of influences upon participants’ ways of thinking about the SDH and the public health role in addressing them: personal upbringings and backgrounds, educational backgrounds and training, and professional experiences.

### Personal upbringings and backgrounds

In many cases, respondents spoke of how their upbringings and backgrounds had come to influence their thinking about societal issues of inequality and inequity and, eventually, the SDH. These included being members of particular classes or racial groups and being exposed to parents’ attitudes and values. These influences occurred prior to their post-secondary education and training. In many cases these influences shaped their choices of post-secondary education and areas of training. The concepts learned during post-secondary education further reinforced their thinking about societal issues of inequality and inequity. These influences were more likely to be spoken of by lead staff than MOHs.

I grew up in a low income household and as an adult I am able to reflect upon some of the influences in my life that led me to be a social worker and then to do community development work. I think I have a personal understanding of what poverty means especially when you’re a child growing up with it so certainly that would be a key influence. -- Staff person

I guess the growing up and coming from a blue collar family. So I’m from the North and my father worked for a mining company so I would have been aware of union issues, about mining incidents, about union versus management kinds of things, and about the health impacts of shift work, about a single industry town and the impact when the company downsized from 30,000 employees to 10,000. -- Staff person

I grew up in [Caribbean nation] and my father was very involved in the church. He was a minister so we practiced and saw it every day. And when I came here and I heard the term social determinants of health it just kind of triggered that hey, this is something that growing up you watched happen, you experienced firsthand and this is just a westernized way of framing and naming an issue that has existed and has persisted in society all along and really has never really been addressed in a concerted way to support people in achieving and getting to their full potential. -- Staff person

I’m not an ethnic majority and certainly, you know, having encountered, a lot of different people, certainly issues of racism, and a general belief that people from single parent families, non-white families would be less successful in life. I think I’ve had more of a sensitivity to some of these issues than perhaps some of my peers. -- MOH

Respondents make the connection between how their ways of thinking about society -- shaped by their background and upbringing -- influence their thinking about the SDH. This should not be surprising as writers have commented on how personal worldviews come to shape attitudes towards health in general and means of promoting health in particular [15-17]. Nonetheless these issues are seldom mentioned in the SDH implementation literature. The next section examines how training and education build upon these earlier experiences and influence ways of thinking and acting on the SDH.

### Educational background and training

MOHs are first trained as physicians and usually complete a residency in *Public Health and Preventive Medicine*. All of the nine MOHs in this study followed this path and referred to this post-medical school training as *Community Medicine*, the title used for this specialty until five years ago. (Some MOHs in Ontario have not completed a Public Health and Preventive residency but instead one in Family Medicine, which may be supplemented by a one year Masters of Public Health. This alternative path usually occurs in rural public health units and, as mentioned, did not apply to participants in this study).

It is not surprising that medical training was not seen as a source of SDH concepts but it is rather striking that none of the MOHs mentioned exposure to SDH concepts during their community medicine training as it is the one medical speciality where such concepts should be found. This is not to say that these concepts were not there, but that it did not resonate for them or influence their thinking about the SDH.

In two cases, MOHs emphasized their learning about the SDH through personal reading spurred on by the increasing profile of SDH in the public health sphere. These MOHs were associated with service delivery-oriented PHUs.

I’ve read the SDH book edited by Sir Michael Marmot. I have read the executive summary of the WHO’s Commission on the Social Determinants of Health. I have read all of the Senate Standing Committee’s reports on population health. And I’ve read other literature that has come my way to get my head around the SDH. – MOH

I’m a Medical Officer of Health. If I don’t think about it I’m not doing my job for one, right? Secondly I did actually do a lot of reading on this fairly recently because I wrote a chapter for a book that the group is bringing out on social epidemiology so I took that occasion to do a lot of reading. -- MOH

As will be shown below, in many cases lead staff respondents indicated their educational training shaped their views on the SDH. For the MOHs however, such exposures seemed to be sporadic and were a result of the individuals’ personal interest in moving beyond basic medical studies by taking courses in sociology and philosophy.

I think certainly in my medical education I was exposed to some people who had a sociological approach to medicine. I remember some of the elective courses and the courses that most medical students don’t care about much. History of medicine and sociology of medicine were of particular interest to me and I had some influential teachers at that stage. But in medical school you’re being streamed into a clinical discipline, fairly directly so those ways of thinking about health really re-emerged later on after I’d been in clinical training for a number of years. -- MOH

Several of the MOHs who were more active in addressing the SDH spoke of exposure to Aboriginal health issues during medical training as shaping their ways of thinking. These experiences combined with their residency in Community Medicine (now called Public Health and Preventive Medicine) to set their path of concern with the SDH.

I worked on a reserve in Western Canada when I was a medical student in a nursing station and saw 12 people in a one room house. You know, the community I was in had one of the highest TB rates in the world. So it’s something I’ve lived with for a long time so I see it everywhere. In local public health work here you can’t name a public health issue that doesn’t have a social determinants of health overlay. – MOH

As part of the residency I probably spent in total almost six months working on Native reserves. So what everybody talks about with Native reserves it kind of slays me how so many people have no idea what is going on up there or they’re totally shocked when they see the stories. I guess because I would have lived it and seen firsthand what basically is happening. – MOH

It appears that many of those who hold the most power in directing PHU activities are provided with a limited academic understanding of the SDH. While education can be very influential, lived experience and/or direct exposure may be more powerful motivators for change.

For lead staff, training in community nutrition, nursing, social work, and international development studies shaped their thinking about the SDH. Many of them were first exposed to the SDH during their undergraduate degrees in nursing, social work, and nutrition. All of the lead staff had graduate degrees and these were in community nutrition, social work and public policy, international development, political science, health promotion, epidemiology, and nursing. It is noteworthy that respondents suggested that their ability to do community-based and policy-related SDH work was a result of their graduate studies in non-public health areas.

And from my perspective I think it’s really from the educational preparation that I’ve had, which has been primarily from a nursing background perspective and obviously with a focus and experience and education within the public health field. As well I would say it’s my own experience from a personal perspective in growing up and exposures to different environments, family setting, community experiences that have contributed to my perspective and how I think about determinants of health at this time. -- Staff person

I think certainly a background in international development introduced a lot of the concepts of the importance of some of the bigger influences on people’s lives, the political and economic influences shaping lives. – Staff person

But I think when I went for a graduate degree in social welfare and focused on community development work I really started to understand. I did placements at the housing health centre. I started to understand how everything is connected so you can’t really look at one service or one program in isolation of how it interacts with everything else going on in people’s lives. I came to understand how as government we make things difficult for people by not connecting the things that will help together. – Staff person

So my Masters is more broadly in organization and leadership transformation and community development, but my research project for my Masters was around the social determinants of health and public health. – Staff person

Interestingly, lead staff – especially those employed in intersectoral and community-focused and public advocacy/public education PHUs -- came from areas outside of public health. They saw their exposure to broader concepts common to social work, political science, social welfare and international development as enabling them to contribute to PHU’s work on the SDH.

Education and professional training contribute to ways of thinking about the SDH. For MOHs, the curriculum content in medical school provided little concerning these issues. It was the specific exposure to Aboriginal health issues of some MOHs, either during medical school or community placements and community medicine residency, that shaped their views. Staff people cited their undergraduate and graduate training and their personal experiences as having influenced their views on the SDH, but it is of note that so many staff with managerial responsibilities for addressing SDH come from outside traditional public health training. They became involved in PHU action on the SDH because the PHUs saw their varied backgrounds as having the potential to contribute to SDH activities.

### Professional experiences

Once employed, some respondents indicated their workplace milieus shaped their thinking about the SDH. Lead staff members entering PHUs where the SDH were treated as a priority expanded their activities to working with coalitions to address broader issues of the health and well-being of communities. In some of these cases respondents moved from the social services and municipal government sectors into public health. This was a result of increasing PHU concern with SDH and a realization that those with training and experience in disciplines with a broader perspective on community and policy would be able to contribute to SDH-related activities.

Well, I’m actually a social worker so I came to the social determinants of health long before I came to public health. I used to work at the social planning council and it was the Lalonde Report that first stimulated talk about social determinants of health in Ontario. The social planning council had tried to incorporate social determinants of health into the work it was doing and tried to connect what at that time was our district health council was doing in the health sphere with what we were doing in the social sphere. – Staff person

When this opportunity came up and I applied for the job it was for a nutrition and physical activity health promoter position. At the end of the interview I was simply asked ‘Is there anything else you would like to add?’ and I went on my little rant about frustrations at being unable to address social and economic conditions. And little did I know that that was actually very up and coming, front and foremost in the mind of the MOH and that was where the unit was moving. – Staff person

In one case, a staff member was required to narrow their approaches as a result of the PHU approach towards the SDH.

I think in public health we’re still very much looking at the behaviour even though we understand other factors influence determinants of health. When you look at our outcomes, when you look at our indicators, you look at what we’re accountable for, I think we’re still what motivates us is the behaviour, the behaviours. – Staff person

Professional experiences can combine with backgrounds and previous experiences to either enhance or limit the likelihood of acting on SDH issues. The following section explores these findings.

Our findings reveal that the manner in which a public health unit addresses the SDH is clearly associated with the personal characteristics and backgrounds of MOHs and the lead staff they hire to carry out these activities. MOHs associated with service delivery-oriented PHUs had little to say about their personal experiences and backgrounds. In contrast, MOHs whose units are identified as intersectoral and community-focused and/or public policy/public advocacy spoke of how their thinking about the SDH have been influenced by their own values and experiences, particularly working with Aboriginal communities in Canada. MOH’s associated with public policy/public advocacy are distinguished by their structural analysis of SDH issues and their belief that PHUs need to take a leadership role in shifting how society thinks about these issues.

Another finding is the extent to which lead staff people responsible for addressing the SDH in the PHUs carrying out intersectoral and community-focused and public education/public policy advocacy came from disciplines outside the traditional public health arena. This is particularly noteworthy in the case of the two PHUs which have taken on a public education/public policy advocacy stance. In these cases, the lead staff people have graduate degrees in political science and social work. In two of the three PHUs that, to date, have limited themselves to service delivery-oriented SDH, the lead staff people have been trained in public health epidemiology and health promotion.

This does not necessarily imply their educational backgrounds determine PHU approach, but it does suggest that their employment is consistent with their training. It is particularly interesting to note that lead staff members in the intersectoral and community-focused PHUs are all trained in community-related activity such as community nutrition and social work and social policy. For these PHUs, their experience in community action has been applied in SDH-related activities. In terms of our critical realist approach, personal exposure and lived experience of structural inequities can be seen as activating features that enable the structures and processes associated with the *real* to become the *actual.* These activating features result in more advanced SDH-related activities, the *empirical*.

### Jurisdictional characteristics and SDH activities

PHUs in Ontario differ on a range of characteristics that can potentially affect how they approach the SDH. As shown in Figure 2, PHUs differ on whether they are regional versus urban, the geography of their jurisdiction, organization of the Board of Health that directs their activities, populations they serve, local politics, and the presence of community organizations with concerns related to the SDH. These categories emerged during inquiries into barriers and supports for their SDH-related activities. We found that many of these issues related to a jurisdiction’s categorization as either “regional” or “urban”.

### Regional vs. urban jurisdiction – with a relationship to geography

Among the nine PHUs involved in the study, three are urban units and six are regional. Regional PHUs are responsible for a wider geographical area that usually contains one or two small cities and a number of smaller towns and villages. Urban PHUs are responsible for a single city. We found that regional PHUs are faced with challenges related to the nature of their Board of Health, wider geography, and fewer community agencies concerned with SDH-related issues. Local politics can also create challenges. We outline some of these challenges below.

Regional PHUs are faced with a challenge in that they are required to address public health issues across a range of communities. This usually involves significant geographical areas and a number of more or less autonomous communities. In contrast, urban PHUs have the ability to focus on a single administrative district in relation to their public health work.

Keeping in mind too that our catchment area is really quite huge geographically so I think within our catchment we have between 16 and 19 municipalities. – Staff person

You’re trying to do things that are right for [name of the region] but we have over ten local municipalities and all these municipalities see themselves as separate and distinct. So I’d say there’s a sense of disconnection and the geography does not serve as well. We have three separate United Ways. So we have a United Way for what’s called […] and area, one that’s […] and district, and I believe there’s one in […] But to me that speaks to this disconnect. We really should have one United Way that really focuses on the entire community. But politically we also have, you know, more than ten separate mayors who have their own agenda, trying to meet their own community needs. – Staff person

Regional PHUs see themselves as facing larger geographical challenges such as coordinating and partnering with multiple and siloed services, serving communities with different demographics, and working with a large number of politicians – who may not necessarily work easily with one another. Urban PHUs did not mention these types of challenges. These challenges can act as barriers to SDH activity because intersectoral collaboration is more difficult to coordinate. Action on the SDH requires support and buy-in from a greater number of decision-makers, and the number of jurisdictions served by the PHU and their variation in social needs can make it challenging to establish SDH-related priorities.

### Board of health organization

The Ontario public health system consists of 36 local public health units of which 22 are autonomous bodies with the remainder having municipal – usually regional -- councils acting as the Board of Health. Autonomous Boards of Health usually consist of both elected representatives and members of the public. Regional PHUs usually do not have a distinct Board of Health associated with the PHU and this is the case for the six regional PHUs in this study. Members of these Boards may be elected politicians or municipal officials and these regional PHUs report more challenges to addressing the SDH than do the urban units with autonomous Boards. In some of the urban PHUs, the Board has stimulated SDH activity. In most other cases, efforts have been undertaken to build support among Board members for SDH-related activities. These efforts have faced mixed results. The first two quotations below are related to having a regional Board, the next three to having an autonomous one.

We have a regional system so our 31 councillors form our Board of Health and most of them were elected on a mandate of fiscal responsibility and restraint. So when we raise these issues, we’ve changed the way that we’ve presented social determinants of health issues. We don’t use the term social determinants of health really anymore. We’ve moved on to using language like making […] a healthy place to live, work, grow and play regardless of your income or your education level, trying to come about it that way. Because addressing the social determinants is seen as sometimes superfluous as compared to some of the other things we do such as restaurant inspections. -- MOH

And the other thing I would add and this is one of the advantages of being part of a regional government, and that is the strong links with our social community service department and the other departments that are around the table such as planning for example. And, and not to mention finance department, public works. By being the head of the health department I get to sit on the senior, what’s called the management committee team for […]. We meet. Meetings can be scheduled weekly. We don’t meet all the time weekly but because of that, you know, your line about what do we see as our role, so for example affordable housing and childcare spaces, that is one of the primary mandates of one of my sister departments if I can use that word, social and community services. So yes, we’ve got an interest in it but I don’t have to start trying to beat my head against the wall to get somebody thinking about it because I have another department whose primary mandate, one could argue is a significant or more so than even public health in dealing with truly some of the upstream issues if you’re looking at basic housing and childcare as well as income support. -- MOH

So ours is an independent Board of Health. If you look at public health structures some are part of regional government and others are not and so ours is not. It’s an independent corporation but the membership is made up overwhelmingly by municipal politicians appointed by our local municipalities in keeping with a regulation of the Health Protection and Promotion Act. I think it’s actually a superb Board. I’ve worked for a number of Boards in the past. I think this is probably the best Board I’ve ever worked with. I think they’re very supportive of our mandate, very interested in our mission, and our strategic planning has participated readily in strategic planning and are very supportive of social determinants as being a priority within strategic planning and has supported advocacy positions we’ve brought to them before for their support. -- MOH

Having a Board that is strongly committed is a big help. They are pushing me as much as I’m supporting them and, you know, we have been pushed for example on the issue of racialization. A member of the Board has been very outspoken on this and has asked for a number of reports and that’s helped me to move that agenda forward inside the organization. -- MOH

Our Board of Health has been supportive. When we presented our position statement and did a presentation to them on what the social determinants of health were, there was unanimous acceptance of that position statement and an agreement that’s the work we should be doing. -- MOH

Yet even in this respect, there is not a perfect relationship between having a regional Board of Health and difficulty in gaining support for a SDH agenda. One MOH from a regional PHU with an autonomous Board states:

Well the Board has been supportive, you know. The chair of our Board is the executive director of the […] and she’s definitely supportive of this work and is an eloquent speaker and certainly able to share these messages with the rest of the Board. The Board hasn’t been in the way or they’ve been supportive all the way along. - MOH

Generally then, PHUs dealing with regional boards face greater challenges associated with having to deal with the entire regional government rather than an autonomous Board of Health.

### Local politics

Local politics play a role in influencing SDH-related PHU activity. Canada has been experiencing a drift towards conservative market-oriented public policies for some time now. Some PHUs are located in areas that traditionally vote for the social democratic New Democratic Party at the provincial and federal levels and have like-minded elected officials at the local level (municipal elections are non-partisan). Other PHUs are located in areas that elect more conservative candidates at all levels. Generally, regional PHUs are seen by interviewees as having more conservative local politics thereby, experiencing more challenges to addressing the SDH. Politically left-leaning jurisdictions are seen as more supportive of a PHU addressing the SDH as it is consistent with their more collective approach to addressing social issues. One staff member from an SDH-active PHU stated:

And we certainly don’t feel [the conservative drift] locally. As you know, we’ve always been a NDP stronghold in many cases. – Staff person

In PHUs where SDH activity is occurring, local politics requires sensitivity to the framing of issues. MOHs and staff need to frame SDH arguments in a form acceptable to elected representatives and the public.

I don’t think for us [the conservative drift] has had much of an influence, we continue to be involved to the same degree at the grassroots level. We continue to do the same activities. We might look for different opportunities or do things in a slightly different way but we continue to just work away at the local level. – Staff person.

If the government changes in the next provincial election we’ll have conservative governments at all three levels which probably will not help. We’ve had discussions about that internally. You know, how does that affect what we try to do in terms of advocacy? I think there are still some opportunities for progress, but it certainly isn’t as optimistic as if we were facing different leadership. -- MOH

In other PHUs, local politics are seen as a barrier to carrying out SDH-related activities.

The other big thing is, so, we live in a very conservative, a politically conservative environment, and we are considered from a profile we are considered a very valued and credible resource in our community. And we need to maintain that relationship forever as a credible and reliable resource. So then when we start looking at public campaigns and speaking out in a more unified voice we have to do it in a way that doesn’t jeopardize those strategic alliances. – Staff person

I just think given that we are a more conservative society that leads to greater emphasis being placed on the individual as opposed to collectives. So when you’re taking an advocacy position you need to be realistic. A significant vocal minority of Canadians -- and given our electoral system -- a majority of our elected officials -- are fairly cool towards providing approaches concerned with collectives as opposed to individuals. Does it make public health officials more skittish with respect to approaching councils, Boards, MPPs and so forth? I think that all depends. Nobody wants to take forward proposals that you know are dead on arrival and the more conservative a particular setting is the more likely it is that these sorts of approaches are dead on arrival and, you know, you may be reluctant to take things forward. – MOH

These quotations suggest that local politics indeed play a role in PHUs addressing the SDH. It suggests a need in many cases to create messages that frame these issues as being concerned with promoting the health and well-being of the community, rather than explicitly expressing SDH issues in political terms. Politics is therefore a potentially activating or inhibiting factor allowing the *real* to become the *actual* in regards to SDH-related PHU activity.

### Community organizations

PHUs differ in the extent to which they are able to interact with a range of community organizations. Urban public health units are more likely to have strong networks of civil society organizations that are concerned with a range of SDH. Regional PHUs are generally in jurisdictions with fewer community organizations.

I think we have great partners out in the community. We’ve taking on an issue like food security. We’ve worked closely with [name of agency] and [name of agency] and had collaborative efficacy and collaborative program development. So that’s a great support to have partners in the community. – MOH

The community partners have been key. We do have a community that elects people that are generally open to these ideas so political support has been pretty key. I don’t think we would have been able to move or convince our Board to do some things we have if they weren’t open to the ideas to begin with. -- MOH

[Name of person] came to [name of city] and did a presentation to some of our healthy lifestyle staff in 2003. And when he was a keynote speaker at the Social Planning Council Annual General Meeting, the chair of our Board of Health was in attendance. So then the Board requested some follow up and a few months later there was a presentation made to the Board of Health on the social determinants. That introduced the process and we did another presentation to the Board of Health a few months later and then they unanimously passed recommendations endorsing the Toronto Charter for a Healthy Canada. That was the underpinnings of the organizational support and it built from there with our social determinants of health committee and then a staff position. But it was an important catalyst to get the discussion going. -- Staff Person

Well we had a mayor who was elected in 2006 who formed an action committee on poverty reduction. So the mayor and a couple of the councillors were very interested and active in this area and took a leadership role so that certainly helped to bring the community together. It gave the health unit and our staff lots of opportunities to participate in the municipal process. So also there are really active social justice groups in [name of city] and very active community agencies and because [name of city] is fairly small the agencies really develop quite close and personal trusting relationships among themselves. It’s quite easy and accepted for multi-agencies just to call each other and get together and discuss an idea or a gap and try and brainstorm around how they can do something about it and then put it into place. It’s not really a big bureaucratic issue to get some of these initiatives started and that’s been really helpful. – Staff person

So I bet you we share the same barriers with [name of other regions] and all those places, right? These are rapidly growing communities and civil society is grossly under-developed, right? I bet [name of city] has 50 times as many NGOs as we do. It’s just not that kind of place. There’s very, very little out there. And if you do get an NGO it’ll either be serving a tiny, tiny sector or it’ll be, you know, completely inadequate to serve a large population. And particularly in the ethno-cultural community it’s just dozens and dozens of little tiny organizations, not necessarily cooperating with each other. And you’ll find that education and social services are as under-funded as health services. So it’s very difficult. You think of [name of city] and everything is the opposite. -- MOH

In short, local environments and the presence of supportive agencies have an influence upon PHUs’ SDH activities. The increasingly popular concept of *intersectoral action* has come to prominence as a means of promoting SDH-related activity but has tended to focus narrowly on health-related interventions by various governmental departments rather than the kinds of broader relationships with various civil society organizations identified here [18]. The findings here suggest attention be directed toward how these collaborations may represent, in part, a building of social movements whose aims extend beyond SDH concepts to a broader concern with societal social justice and equity [19].

These relationships do not come naturally. Whether the local environment is seen as supportive or not, the PHU has to work actively to build trust with local agencies.

The public health unit has spent a lot of time nurturing relationships with all of those groups and so most of the relationships we have with organizations are based in a history that is not specifically the social determinants of health. For example with the YMCA, the background would be around things like physical activity and having shared space with them with childcare centres and early learning centres. Other groups such as the United Way have relationships with the unit by way of one of the associate medical officers of health and our medical officer chairing United Way campaigns. And I think that that has been really positive because of the long time trajectory around addressing the social determinants of health with its ups and downs allow you to weather those downs together and it doesn’t mean an end to the relationship. I also think that in the past we’ve had some very strong political leaders who have been extremely supportive of social determinants of health issues. -- MOH

Jurisdictional characteristics therefore also play a role in whether and how PHUs address the SDH. In urban PHUs the geography and type of Board facilitates movement from the *real* to the *actual* while the opposite is the case for regional PHUs. MOHs and staff are sensitive to local politics and ascribe an influence to this as well.

## Discussion

The characteristics associated with how PHUs approach the SDH interact in complex ways. It is difficult to isolate the specific characteristics of jurisdictions that shape the PHUs activities, but after reviewing our findings we identify five distinctive groupings that illustrate how jurisdictional characteristics that have been discussed in the previous section are associated with PHU SDH approach. Table 1 provides a summary of these findings.

**Table 1:** **Groupings related to local structures and environments and PHUs’ SDH activity**

*Public Education and Public Advocacy*	
*Regional (1 PHU)*	*Urban (1 PHU)*
Urban centre within larger geographical area.	Urban jurisdiction.
Well developed network of social service and support agencies within the urban centre.	Well developed network of social service and support agencies.
PHU reaches out to these agencies and provides both leadership and support.	PHU reaches out to these agencies and provides both leadership and support.
Progressive political environment.	Progressive political environment.
Board of Health provides both impetus and support for SDH-related action.	Board of Health provides both impetus and support for SDH-related action.
*Intersectoral and Community Focused*	
*Urban (2 PHUs)*	*Regional (2 PHUs)*
Urban jurisdiction.	Regional jurisdiction spread over larger area.
Well-developed network of social service and support agencies.	Undeveloped network of social service and support agencies.
PHU reaches out to these agencies and provides support for intersectoral action.	Works primarily within regional government structures and participates with networks.
Progressive political environment.	Conservative political environment.
Board of Health is supportive but does not provide impetus for action.	Board of Health consists of entire regional council and has been brought along on issues.
*Regional/Service Delivery-Oriented (3 PHUs)*	
Regional jurisdiction spread over larger area.	
Undeveloped network of social service and support agencies.	
PHU has not yet reached out to social service and support agencies.	
Conservative political environment.	
Board of Health consists of entire regional council and has not been concerned with these issues.	

### Public policy and public education

The two PHUs carrying out public policy advocacy and public education differ in that one is an urban unit while the other is regional. They are similar in that they report numerous supports to their SDH-related activities and these supports are primarily located in an urban centre (the regional unit has a significant city in its jurisdiction). Their supports include a responsive political environment and a network of supportive community agencies working on SDH-related activities. Agency supports are a result of the PHU reaching out to these agencies and providing both leadership and support for a SDH agenda.

In both cases, the stand-alone Board of Health consists of both elected officials and citizen members and has been supportive of the PHUs’ SDH thrust. Again, this is the result of the PHU expending significant efforts to educate Board members. The primary driver of SDH activity is the MOH and lead staff’s belief in the value of this agenda. They then identify the means to facilitate this thrust. The PHUs exercise central control over their SDH efforts with evaluation and accountability across the unit being the responsibility of specific assigned lead staff.

For those who see public health as having a central role in changing how a society thinks about promoting health through action on societal structures and processes, these PHUs provide a working model of how to go about it.

### Intersectoral and community-focused PHUs

Two of the intersectoral and community-focused PHUs are urban units and two are regional. In the urban units, the two cities have a well-established network of social service and support agencies. The PHUs reach out to these agencies and provides support, but have made an explicit decision not to provide leadership in public policy advocacy and public education for these networks. This appears primarily to be a reflection of the ideological beliefs of the MOH and lead staff who take an opportunity-based approach where SDH are seen as limiting people’s opportunities that can best be addressed though community-based work and not public health.

Both of the urban, intersectoral and community-focused PHUs report to a stand-alone Board of Health that is supportive of the SDH-approach. At the City Council level, one unit reports a positive supportive environment while the other reports a more mixed picture. However, it appears these PHUs have not taken full advantage of this support to move towards a leadership role in public policy advocacy and public education regarding the SDH, as is the case for the two public policy and public education PHUs described above. These PHUs do not exercise centralized control over SDH-related initiatives but devolve responsibility for implementation and accountability to PHU departments or units.

The two regional PHUs are spread over a larger area that includes smaller cities, towns, and villages. The network of social service and support agencies is less developed than those of the urban PHUs. Intersectoral work is facilitated at the regional government level where the Board of Health consists solely of elected representatives. There is some participation with regional networks concerned with promoting the region’s well-being. The political environment appears to be more conservative than in the urban centres. Nevertheless, the PHUs are pushing forward with their agenda and seeking to build support. These two PHUs also do not exercise centralized control and devolve responsibility and accountability to PHU departments or units.

These PHUs provide a model by which public health can collaborate with existing sectors to promote health through reform of existing institutions and agency efforts. The approach has the potential to influence public policy change, but it does not place public health at the vanguard of such efforts.

### Service delivery-oriented PHUs

These three PHUs are all regionally organized with responsibilities for numerous smaller cities, towns, and villages. The network of social service and support agencies is generally less developed and the PHUs have not reached out to social service and support agencies to develop a SDH agenda. These PHUs operate within a generally conservative political environment. The Board of Health is generally absent from discussions about SDH work. The MOHs have a functional view of the role that PHUs should play in addressing the SDH, which is very much a reflection of their personal backgrounds, experiences, and ideologies, as well as the jurisdictional environments in which they operate.

These PHUs provide a model of the traditional approach to providing health-related services and programs to disadvantaged populations. It recognizes the important role that the SDH play in shaping health and the importance of providing these disadvantaged sectors with responsive and appropriate supports and services.

## Conclusions

These findings suggest that translating the potential for SDH-related activities into action is very much a result of the complex interaction of ideological beliefs held by the MOH influenced by local jurisdictional features. In terms of our critical realist approach, at the level of the *real* there would appear to be sanction for PHUs in Ontario to address the SDH in a variety of ways. However, this sanction is ambiguous at best as there is no requirement or exposition of how PHUs are supposed to do so. Additionally, since MOHs are hired by local jurisdictional authorities, it is understandable that many would not want to go out on a limb in carrying out activities that will not be understood or well received by such authorities. As noted earlier, no senior level of government in Canada – Federal, provincial, or territorial – has made addressing the SDH an explicit goal of their ruling agenda. Mandating the addressing of the SDH by local authorities in a variety of ways in the Public Health Standards might go a long way in strengthening the structures and processes available for addressing the SDH by local PHUs.

At the level of the *actual*, facilitators of action include: an autonomous Board, being an urban jurisdiction, a socially progressive political environment, and presence of supportive community agencies. However, carrying out SDH-related public health activities requires a firm belief that such efforts are useful and a commitment to carry out the work necessary to see this work brought to fruition. This process will involve educating members of the Board of Health and local elected representatives, reaching out to agencies whose mandate is related to various SDH, and a willingness to help shift the status quo.

We have suggested elsewhere that the ideological beliefs held by MOH are the primary drivers of SDH-related action and findings from this analysis do not dispute this [5]. This article adds to our earlier work by revealing that the features that can activate existing structures and processes to address SDH issues require aligning a range of factors that include personal characteristics and jurisdictional features. These efforts may be more difficult in PHUs that are responsible for a region as opposed to a city and in more politically conservative than progressive areas. Attempts to meaningfully address the SDH will only take place when the PHU indicates a commitment to carry out these SDH activities – or such activities are institutionalized within the organization.

The current situation, whereby initiative on the part of local PHUs is the primary driver of SDH-related activities, is a reflection of the lack of guidelines by the Ministry of Health and Long-Term Care on how to carry out these activities. As one MOH states:

The Public Health Standards themselves have been a bit of a barrier because they weren’t specific in requiring specific actions and accountability. A sort of check for what every health unit should do in terms of social determinants. As a result, the other items are much more specific in the Public Health Standards, and this forces units to attend to those and they’re so busy meeting some of those standards and objectives that it’s hard for them to really make progress upstream on those issues. – MOH

As a result, it is not surprising that PHU action is a function of the micro- and meso-level characteristics we have identified. It appears that the easiest way to facilitate the activation of structures and processes (*the real*) into action (*the actual*) that produces the kinds of SDH activities we have described (*the empirical*) is for the Ministry of Health and Long-Term Care to mandate such action at the macro-level. This view would not be inconsistent with the principle that local public health efforts should be context-driven and tailored to meet local needs, but it would serve to shift these local efforts to a higher plane of action. If such a mandate came about, the issues we have identified would be the factors that PHUs need to take into account as they carry out their required mandate of addressing the SDH.

From a research perspective, there is a need to extend inquiry into exploration of the micro-level (individual attitudes and perspectives), meso- (jurisdictional and organizational) and macro-level (Ministry and provincial government mandates and directives) influences that shape SDH action at the provincial level. How do civil servants at Ministries of Health and related ministries understand these issues? How do they feel bound by elected officials and public understandings of these issues? What do they see as means of moving the SDH agenda forward?

The generality of our findings is limited by our focus on the Ontario scene. PHUs in Ontario operate on a governance model that is very different from the rest of Canada, where public health is integrated into regionalized health authorities. As such there is no official distinction between ‘regional’ and ‘urban’ jurisdictions in those places and PHU activity is more likely to be linked to broader health policy considerations than may be the case in Ontario. Whether these features make it more likely that SDH-related issues will be addressed by local PHUs is unclear and deserves inquiry.

Our findings indicate that action on the SDH by local PHUs is not a simple result of knowledge creation and dissemination. It is a result of a complex interplay of micro-, meso- and macro-level factors that involves recognition of the contested nature of public health, Ministry of Health mandates, and politics. A critical realist perspective recognizes these complexities and should be a useful approach for understanding these issues.

## Abbreviations

JWG: Joint Working Group on the Social Determinants of Health
MOH: Medical Officers of Health
PHU: Public Health Unit
SDH: Social Determinants of Health
